# Roux-En Y Gastric Bypass Surgery Induces Genome-Wide Promoter-Specific Changes in DNA Methylation in Whole Blood of Obese Patients

**DOI:** 10.1371/journal.pone.0115186

**Published:** 2015-02-24

**Authors:** Emil K. Nilsson, Barbara Ernst, Sarah Voisin, Markus Sällman Almén, Christian Benedict, Jessica Mwinyi, Robert Fredriksson, Bernd Schultes, Helgi B. Schiöth

**Affiliations:** 1 Department of Neuroscience, BMC, box 593, 75124 Uppsala, Sweden; 2 Interdisciplinary Obesity Center, eSwiss Medical & Surgical Center, St. Gallen, Switzerland; Scientific Directorate, Bambino Hospital, ITALY

## Abstract

**Context:**

DNA methylation has been proposed to play a critical role in many cellular and biological processes.

**Objective:**

To examine the influence of Roux-en-Y gastric bypass (RYGB) surgery on genome-wide promoter-specific DNA methylation in obese patients. Promoters are involved in the initiation and regulation of gene transcription.

**Methods:**

Promoter-specific DNA methylation in whole blood was measured in 11 obese patients (presurgery BMI >35 kg/m^2^, 4 females), both before and 6 months after RYGB surgery, as well as once only in a control group of 16 normal-weight men. In addition, body weight and fasting plasma glucose were measured after an overnight fast.

**Results:**

The mean genome-wide distance between promoter-specific DNA methylation of obese patients at six months after RYGB surgery and controls was shorter, as compared to that at baseline (p<0.001). Moreover, postsurgically, the DNA methylation of 51 promoters was significantly different from corresponding values that had been measured at baseline (28 upregulated and 23 downregulated, P<0.05 for all promoters, Bonferroni corrected). Among these promoters, an enrichment for genes involved in metabolic processes was found (n = 36, P<0.05). In addition, the mean DNA methylation of these 51 promoters was more similar after surgery to that of controls, than it had been at baseline (P<0.0001). When controlling for the RYGB surgery-induced drop in weight (-24% of respective baseline value) and fasting plasma glucose concentration (-16% of respective baseline value), the DNA methylation of only one out of 51 promoters (~2%) remained significantly different between the pre-and postsurgery time points.

**Conclusions:**

Epigenetic modifications are proposed to play an important role in the development of and predisposition to metabolic diseases, including type II diabetes and obesity. Thus, our findings may form the basis for further investigations to unravel the molecular effects of gastric bypass surgery.

**Clinical Trial:**

ClinicalTrials.gov NCT01730742

## Introduction

An intervention that effectively reduces body weight in obese humans is Roux-en-Y gastric bypass (RYGB) surgery [1, 2]. This procedure reduces the functional volume of the stomach, and thereby significantly shortens the gastric residence time of food. In addition to its pronounced weight-reducing effects, RYGB surgery has been found to counteract many obesity-related metabolic abnormalities [1], including an impaired glycemic metabolism [3] and type 2 diabetes [4].

Interestingly, variations of body weight and plasma glucose concentrations have previously been associated with fluctuations in DNA methylation in humans, including promoter sites [5]. Promoters are DNA regions upstream of a gene that is typically involved in the initiation and regulation of gene transcription. DNA methylation represents an epigenetic mechanism that regulates gene expression in human cells [6], and an aberrant DNA methylation profile might contribute to many pathophysiological conditions observed in obese humans (e.g. [7], [8]). Specifically, weight loss induced by both RYGB and a very low calorie diet (VLCD) has been associated with promoter methylation changes in several genes.[9]

Against this background, the present study aimed to examine whether RYGB surgery has an impact on DNA methylation of gene promoters. To this study aim, genome-wide promoter-specific DNA methylation in whole blood was measured twice in 11 obese patients, both before and 6 month after RYGB surgery. In order to explore if RYGB surgery would reduce the genome-wide distance between promoter-specific DNA methylation of obese patients and normal-weight humans, we also sampled whole blood from 16 normal-weight men.

## Methods

### Patients

**Fig 1:**
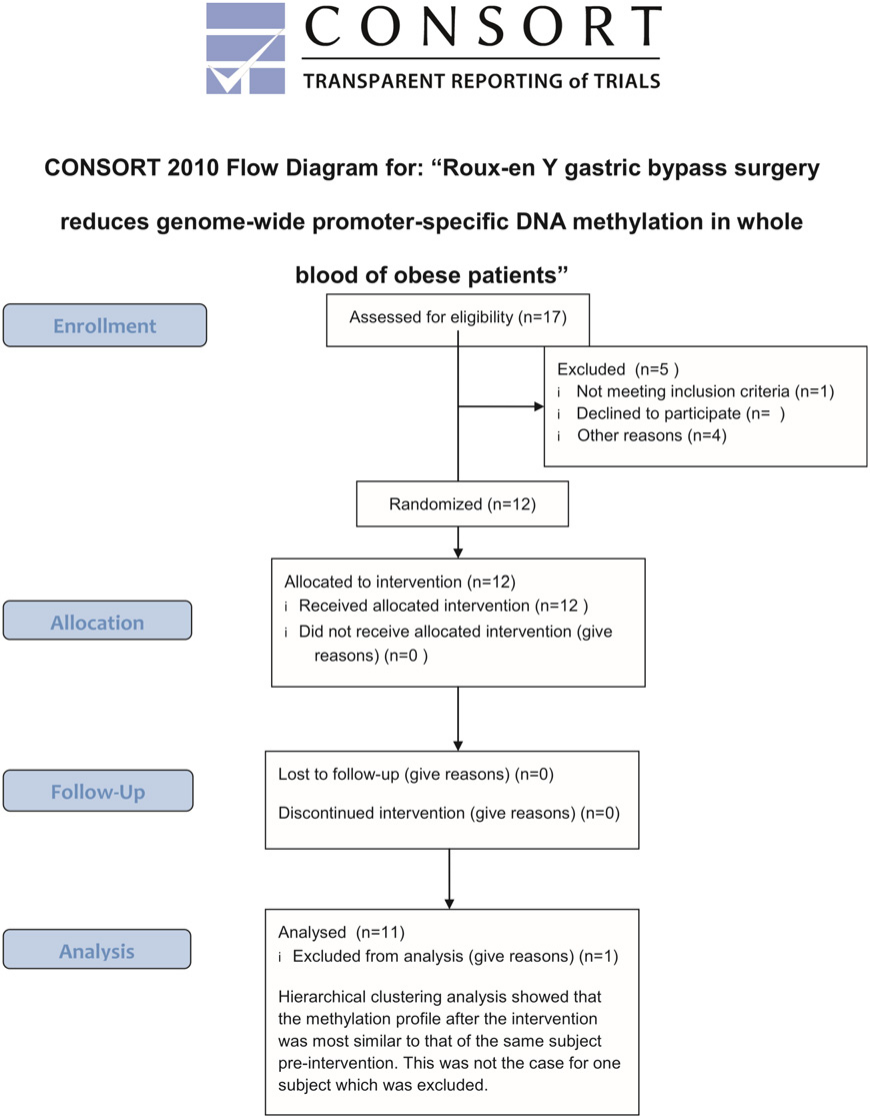
Consort flowchart detailing how the subjects were rectuited and handled throughout the study.

Eleven obese patients who qualified for bariatric surgery according to international guidelines [10] and were willing to undergo a RYGB procedure were included in the study. Patients were recruited in to the study at their time of surgery between 20090820 and 20110210 and were followed up 6 months after the surgery. Thus patient follow-up was performed February 2010 for the first patient and August 2011 for the latest patient. Individuals included in the St Gallen cohort have been well characterized with regard to medical history, medical diagnoses and the intake of any prescribed medications. Subjects, who were included into our genome wide studywere selected from the entire cohort based on two criteria. Individuals should have been only treated with therapeutics that pertained directly to the surgical intervention, e.g general anesthesia. Furthermore, no medical diagnoses other than obesity were allowed to be abundant. Written informed consent was obtained from all subjects, and the study was approved by the ethic committee of Canton St. Gallen, Switzerland. Ethical approval for the study was obtained 20080717. A consort flowchart of the selection procedure is available as Fig. 1 along with the Trend checklist (S1 Trend Checklist). The control group comprising 16 normal-weight men was obtained from the control condition of an as of yet unpublished study (Clinical trial number: NCT01730742). This study was approved by the regional ethic committee in Uppsala (regionala ekitprövningsnämnden i Uppsala, www.epn.se), Sweden and written, informed consent was signed by all participants. At the time of recruitment, prospective registration of intervention studies was not the norm and the authors opted to not register the study and ethical approval from committee of Canton St. Gallen and the regional ethical committee at Uppsala University was deemed sufficient. The authors confirm that all ongoing and related trials for this intervention are now registered.

### RYGB Procedures

During the RYGB procedure the largest part of the stomach was transected, and a small gastric pouch of about 20–30 ml was then anastomized to the proximal jejunum with the diameter of the pouch–jejunal anastomosis standardized to be about 12 mm. In addition, the biliopancreatic limb is side to side anatomized to the jejunum 150 cm distal from the pouch–jejunal anastomosis (Roux-en Y limb length, 150 cm).

### Assessments

For the determination of promoter-specific DNA methylation, whole blood was sampled after an overnight fast in EDTA coated tubes, both at baseline (~1–2 weeks before the surgery) and six months after RYGB. In the control group, whole blood was sampled in the morning after nocturnal sleep (data are not shown). Until assay, all whole blood samples were kept frozen at -80 C. Weight was measured while subjects were wearing light close. Fasting plasma glucose (FPG) levels were measured by routine clinical laboratory analyses.

### Methylation profiling

Genomic DNA was extracted using the phenol-chloroform method [11]. Bisulfite conversion was performed with the EZ DNA Methylation—Gold kit (Zymo research. Cat. No. D5004). The degree of DNA methylation was determined by the Illumina 450K methylation chip (Illumina, San Diego, USA). After the bisulfite converted DNA was hybridized to the array, fluorescence was measured using the Illumina iScan system. The raw data were processed using GenomeStudio 2009–2. Blood samples from each subject were hybridized to the same physical chip to minimize biases. Note that one subject of an initial sample of 12 obese humans was not considered eligible for analysis because of technical failure during the methylation profiling procedure.

### Statistical analysis

Beta values were produced by Genomestudio (Illumina, San Diego, USA) and preprocessed using Illumina Methylation Analyzer (IMA) package for R [12, 13]. Probes were excluded if the detection p-value exceeded 5E-5 in 75% or more of the samples. Probes annotated to known snp-sites or sex chromosomes were excluded prior to peak correction and quantile normalization. An average on all CpG sites located in the same promoter region was calculated as this analytical approach is proposed to be more meaningful from a biological perspective [14]. In total, 16724 promoter regions were used for subsequent analysis.

The initial analysis aimed to examine if RYGB surgery would reduce the genome-wide distance between promoter DNA methylation of obese patients and the control group (comprising normal-weight men). To this aim, the Euclidean distance of all promoters (i.e. 16724) was estimated for both *presurgery time point vs. control group* and *postsurgery time point vs. control group*. These distances were then compared by means of a pairwise t test. In the second analysis, presurgery DNA methylation of the above mentioned 16724 promoter regions was compared with that obtained at six month after RYGB (controlled for multiple comparisons, i.e. Bonferroni corrected). All significant promoter hits revealed by this pre/post comparison were then subjected to an additional Euclidean distance analysis. Overall, a p-value <0.05 was considered significant.

## Results

### Descriptive

Descriptive statistics of the participants are shown in Table 1. As expected, following RYGB surgery obese patients reduced their body weight by about 24%. However, patients’ weight remained significantly higher compared to the normal-weight group. Fasting plasma glucose levels were lower at six month after RYGB compared with baseline values (- 16%), with no statistical difference between obese patients at the postsurgery time point and normal-weight controls.

**Table 1:** Descriptive statistics.

	**Subjects at baseline^A^**	**6 months after RYGB surgery^B^**	**Normal-weight controls^C^**	**P-valueA vs. B**	**P-ValueA vs. C**	**P-ValueB vs. C**
	**Mean ± SEM**	**Mean ± SEM**	**Mean ± SEM**			
*Number of subjects*	11	11	16	--	--	--
*Females/Males (n/n)*	7/4	7/4	0/16	--	--	--
*Age*, *in years*	47 ± 4	—	24 ± 1	--	--	--
*Weight*, *in kg*	132 ± 7	100 ± 6	78 ± 2	<0.001	<0.001	<0.01
*Body mass index*, *kg/m^2^*	48.2 ± 2.2	36.4 ± 2.1	23.6 ± 0.6	<0.001	<0.001	<0.001
*Morning FPG*, *mg/dl*	6.0 ± 0.4	5.1 ± 0.2	5.4 ± 0.1	<0.05	0.08	0.09
*Plasma insulin*	14.12 (2.6)					

P-values were calculated by means of t-tests. A one-tailed p-value < 0.05 was considered significant. Abbreviations: FPG, fasting plasma glucose.

### DNA methylation

**Fig 2:**
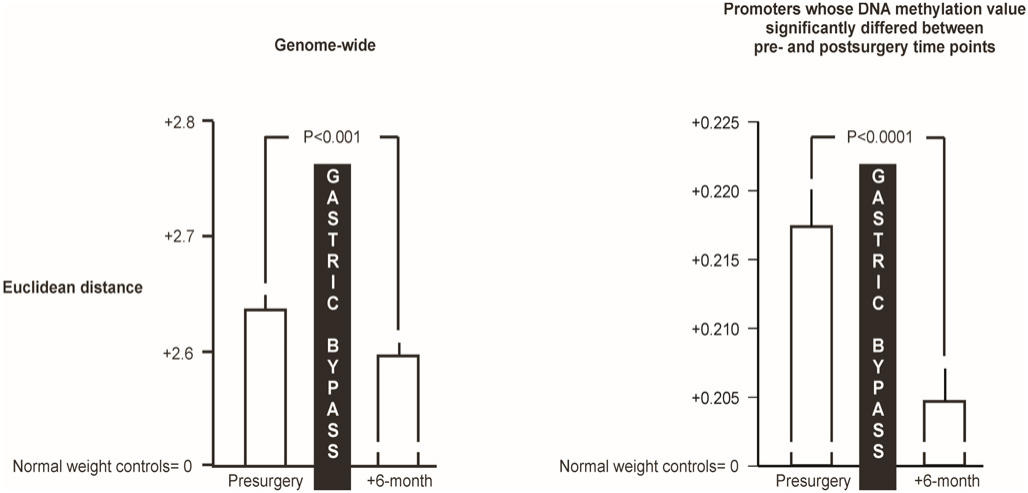
Mean (± SEM) genome-wide Euclidean distance between promoter-specific DNA methylation of obese patients and controls before and at 6 month after Roux-en Y gastric bypass surgery. The left panel illustrates the mean Euclidean distance at genome-wide scale, whereas the right panel shows the mean Euclidean distance at single-gene promoter scale. A two-tailed p-value <0.05 was considered significant.

**Fig 3:**
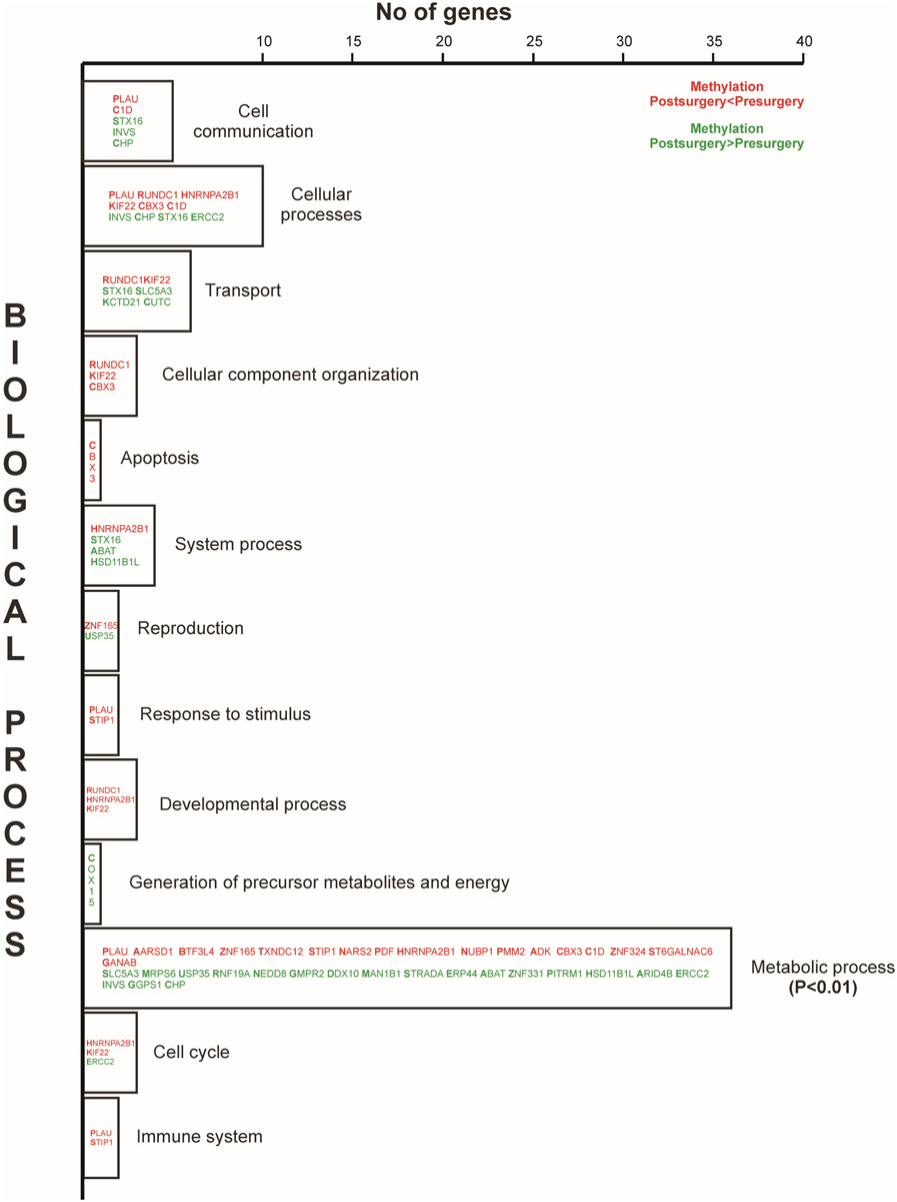
GO terms classification of genes (N = 51) whose promoter-specific DNA methylation was significantly different between baseline (i.e before Roux-en Y gastric bypass surgery) and at six month after surgery. An enrichment analysis of GO-terms biological processes using DAVID functional analysis revealed an enrichment for genes involved in metabolic processes (GO: 0008152) (P<0.05).

The mean genome-wide Euclidean distance between promoters of obese patients at six month after RYGB surgery and controls was significantly shorter, as compared to that at baseline (P<0.001, Fig. 2, **left panel**). An additional analysis demonstrated that at six month after RYGB surgery, the DNA methylation of 51 promoters was significantly different from corresponding presurgery values (28 were upregulated and 23 were downregulated, P<0.05 for all promoters, Bonferroni corrected; Table 2). The mean DNA methylation of these 51 promoters was also more similar after surgery to that of controls, as compared to that at baseline (P<0.0001; Fig. 2, **right panel**). Importantly, when controlling for the RYGB surgery-induced drop in weight (-24% of respective baseline value) and fasting plasma glucose concentrations (-16% of respective baseline value), the DNA methylation of only one out of 51 promoters (~2%) remained significant at the postsurgery time point. An enrichment analysis of GO-terms biological processes using DAVID functional analysis revealed an enrichment for genes involved in metabolic processes (GO: 0008152) (P<0.05, Benjamini-Hochberg correction, Fig. 3).

**Table 2:** Whole-blood promoter-specific DNA methylation in obese patients before and at 6 month after Roux-en Y gastric bypass (RYGB) surgery.

**Gene**	**% DNA Methylation level of the promoter region (Mean ± SEM)**		**P-value**
	**Baseline**	**6 months after RYGB surgery**	
SLC5A3;MRPS6^1^	51.95±2.46	57.78±3.09	1.27E-02
MAN1B1;LOC100289341	3.99±0.54	8.67±0.94	7.10E-04
GGPS1;ARID4B	17.22±0.89	20.57±1.01	1.03E-03
CHP;EXD1	15.83±1.26	19.26±1.57	3.75E-02
LOH12CR1	17.46±1.09	19.17±0.81	1.44E-02
RNF19A	2.54±0.46	4.85±0.81	2.64E-02
SLC5A3;MRPS6^1^	23.66±0.95	25.92±1.24	9.16E-05
C4orf33;SCLT1	23.66±0.34	25.83±0.35	4.26E-02
INVS;ERP44	47.3±0.52	48.97±0.48	4.86E-02
STRADA^2^	49.22±0.87	50.51±0.8	2.24E-02
STX16	3.79±0.26	5.2±0.2	1.60E-03
C9orf93	11.73±0.26	13.25±0.31	5.09E-03
NEDD8;GMPR2	13.37±0.58	14.48±0.21	4.50E-02
KCTD21;USP35	9.5±0.91	10.48±0.6	2.01E-02
SCLT1;C4orf33	15.18±0.2	16.45±0.22	1.97E-02
ABAT	1.94±0.21	2.6±0.18	2.85E-03
ERCC2	4.55±0.24	5.27±0.13	5.30E-04
TRIP11	1.26±0.14	2.04±0.25	2.18E-02
C19orf70;HSD11B1L	95.86±0.26	96.92±0.29	3.04E-02
STRADA^2^	22.87±0.26	23.61±0.21	9.43E-03
ZNF321	5.64±0.69	6.13±0.33	1.93E-02
CTR9	16.81±0.26	17.55±0.27	2.14E-03
DENND1C	16.29±0.45	16.78±0.3	3.98E-03
ZNF331	9.51±0.15	10.22±0.14	1.51E-02
PITRM1	10.54±0.4	10.92±0.25	8.90E-03
KPTN	4.83±0.28	5.41±0.21	4.47E-02
COX15;CUTC	2.2±0.13	2.84±0.26	2.23E-02
DDX10	3.89±0.21	4.43±0.28	4.27E-02
ZNF165	3.31±0.14	2.75±0.17	5.28E-03
PDF;COG8	8.63±0.47	8.05±0.43	2.93E-02
NUBP1	3.49±0.2	2.93±0.14	1.27E-02
GANAB;INTS5	2.58±0.16	2.07±0.11	4.78E-02
PMM2;TMEM186	3.47±0.14	2.7±0.09	2.87E-02
C4orf41;RWDD4A	4.88±0.27	4.22±0.22	1.66E-03
ADK	5.39±0.21	4.86±0.28	2.17E-03
CBX3;HNRNPA2B1	5.41±0.32	4.78±0.2	3.17E-02
C1D	9.63±0.55	8.82±0.46	3.38E-02
TMC6	6.79±0.72	5.8±0.57	1.49E-02
KIF22	7.75±0.27	6.97±0.36	4.38E-02
C16orf46	13.61±0.4	12.91±0.29	1.14E-02
CDK5RAP1	6.15±0.29	5.21±0.18	1.96E-02
RUNDC1;AARSD1	9.95±0.35	8.99±0.24	2.24E-02
STIP1	15.8±0.35	14.36±0.56	1.31E-02
MORC2	10.66±0.48	9.18±0.38	1.41E-02
INCA1	13.47±0.64	11.89±0.34	3.29E-05
TXNDC12;BTF3L4	14.83±0.69	13.5±0.61	8.79E-03
ST6GALNAC6	16.32±0.61	14.81±0.47	2.58E-02
NARS2	13.09±0.64	11.63±0.62	4.53E-04
ZNF324	5.52±0.37	3.9±0.16	1.93E-02
C10orf55;PLAU	23.93±0.65	22.2±0.35	4.62E-03
C1orf86;LOC100128003	41.12±1.55	38.84±1.53	1.82E-03

The table shows gene promoters whose DNA methylation differed between baseline and 6 months after RYGB surgery. Overall, a Bonferroni corrected two-tailed p-value <0.05 was considered significant.

^1^ The difference between the two entries is that they are from two different promoters. One is the promoter for two splice variants (topmost entry); the other is cell type specific and only affects one splice variant of each gene.

^2^ There are two STRADA promoters associated with two sets of splice variants for that gene.

## Discussion

Here we demonstrate that Roux-en Y gastric bypass (RYGB) surgery decreases the genome-wide presurgery distance between promoter-specific DNA methylation in whole blood of obese patients and controls. These findings may form the basis for further investigations to unravel the molecular effects of gastric bypass surgery, as epigenetic modifications are proposed to play an important role in the development of metabolic diseases, including type 2 diabetes and obesity [15].

We observed that the mean genome-wide distance between promoters of obese patients at six month after RYGB surgery and controls was significantly shorter, than it had been before surgery. Notwithstanding, when adjusting for the RYGB surgery-induced drop in body weight and fasting plasma glucose, only one out of the 51 promoters that were significantly different between presurgery and postsurgery time points (following Bonferroni correction) remained significant. This suggests that the surgery-related influence on body weight and fasting plasma glucose may play a causative role for concomitant changes in DNA methylation. Among the promoter probes whose DNA methylation was significantly changed at 6 month after RYGB surgery, several might account for some metabolic and clinical benefits that are typically observed in response to the RYGB procedure. For instance, INCA1, whose methylation was about 12% lower at 6 month after RYGB than it had been at baseline, may have an anti-cancer effect due to its anti-proliferative properties [16]. Interestingly, cancer incidence and mortality data from the *Utah Cancer Registry* have recently demonstrated that gastric bypass results in lower cancer risk [17]. Another example of putatively clinically relevant changes in DNA methylation is the ADK gene, whose methylation was about 10% lower at 6 month after RYGB than it was at baseline. This gene encodes the enzyme adenosine kinase, which serves as a potential regulator of concentrations of extracellular adenosine and intracellular adenine nucleotides. Interestingly, studies with non‐selective adenosine antagonists have shown that adenosine can improve insulin secretion and lower glucose production [18, 19], as well as stimulate glucagon secretion [20]. Thus, respective changes in DNA methylation could very well contribute to the improved in glucose metabolism and remission of diabetes after RYGB surgery [2, 4].

Adding further grist to the mill that RYGB surgery can cause epigenetic changes in obese humans, a recent study has demonstrated that a RYGB-induced weight loss over six months was correlated with widespread changes in promoter-specific DNA methylation in the skeletal muscle of obese patients. Intriguingly, in this study it was also observed that a subset of genes involved in insulin pathways showed an association between gene expression and promoter methylation [21]. This supports the view that RYGB-induced differences in skeletal promoter-specific DNA methylation are biologically relevant, and may thus contribute to the metabolic health benefits that are often seen after RYGB surgery [1, 2, 4].

Several limitations apply to our study findings: They derive from whole blood samples. That said, as long as they have not been validated in future studies, they cannot be extrapolated to differences in DNA methylation that may occur in other tissues in response to RYGB. Furthermore, the reader should take into account that our study is a six-month snapshot of RYGB-induced changes in promoter-specific DNA methylation. This time point after surgery is characterized by a pronounced catabolism and negative energy balance. Thus, caution is warranted when extrapolating our DNA methylation findings to other time points after the surgery, i.e. when the patients have stabilized their body weight. The control group in the present study served as a reference for DNA methylation levels under conditions of normal weight and normal glucose metabolism. Thus, although our study findings indicate that the whole blood DNA methylation of promoters in obese patients goes towards methylation levels of normal-weight subjects at six month after RYGB surgery, they do not allow firm conclusions as to whether promoter-specific DNA methylation per se has been reset to physiological values. Finally, potential confound induced by factors not entered into our analysis cannot be excluded (e.g. variation in DNA methylation induced by changes in environmental light intensity).

In summary, our results show that RYGB surgery concurs with changes in methylation at DNA sites that are typically involved in the initiation and regulation of gene transcripts. Bearing in mind that we did not measure whole-blood gene transcription, an important next step is to investigate if the RYGB-induced differences in promoter-specific DNA methylation in whole blood are associated with differences in gene expression.

## Supporting Information

S1 Trend Checklist The trend checklist lists where the relevant information about ethical approvals and study outlines are found in the main paper. (TIFF)

## References

[1] Buchwald H, Avidor Y, Braunwald E, Jensen MD, Pories W, et al (2004) Bariatric surgery: a systematic review and meta-analysis. JAMA 292: 1724–1737. 10.1001/jama.292.14.1724 15479938

[2] Adams TD, Davidson LE, Litwin SE, Kolotkin RL, LaMonte MJ, et al (2012) Health benefits of gastric bypass surgery after 6 years. JAMA 308: 1122–1131. 10.1001/2012.jama.11164 22990271 PMC3744888

[3] Bojsen-Møller KN, Dirksen C, Jørgensen NB, Jacobsen SH, Hansen DL, et al (2013) Increased Hepatic Insulin Clearance After Roux-en-Y Gastric Bypass. Journal of Clinical Endocrinology & Metabolism. 10.1210/jc.2013-1286 23609835

[4] Dixon JB, le Roux CW, Rubino F, Zimmet P (2012) Bariatric surgery for type 2 diabetes. Lancet 379: 2300–2311. 10.1016/S0140-6736(12)60401-2 22683132

[5] Yang BT, Dayeh TA, Kirkpatrick CL, Taneera J, Kumar R, et al (2011) Insulin promoter DNA methylation correlates negatively with insulin gene expression and positively with HbA(1c) levels in human pancreatic islets. Diabetologia 54: 360–367. 10.1007/s00125-010-1967-6 21104225 PMC3017313

[6] Kouzarides T (2007) Chromatin Modifications and Their Function. Cell 128: 693–705. 10.1016/j.cell.2007.02.005 17320507

[7] Almen MS, Jacobsson JA, Moschonis G, Benedict C, Chrousos GP, et al (2012) Genome wide analysis reveals association of a FTO gene variant with epigenetic changes. Genomics 99: 132–137. 10.1016/j.ygeno.2011.12.007 22234326

[8] Xu X, Su S, Barnes VA, De Miguel C, Pollock J, et al (2013) A genome-wide methylation study on obesity: Differential variability and differential methylation. Epigenetics 8 10.4161/epi.24506 23644594 PMC3741222

[9] Kirchner H, Nylen C, Laber S, Barres R, Yan J, et al. (2014) Altered promoter methylation of PDK4, IL1 B, IL6, and TNF after Roux-en Y gastric bypass. Surg Obes Relat Dis. 10.1016/j.soard.2013.12.019 24837562

[10] Fried M, Hainer V, Basdevant A, Buchwald H, Deitel M, et al (2007) Inter-disciplinary European guidelines on surgery of severe obesity. Int J Obes (Lond) 31: 569–577. 17325689 10.1038/sj.ijo.0803560

[11] Sambrook J FE, Maniatis T (1989) Molecular cloning: a laboratory manual. Cold Spring Harbor Laboratory press.

[12] Team Rc (2012) R: A Language and Environment for Statistical Computing.

[13] Wang D, Yan L, Hu Q, Sucheston LE, Higgins MJ, et al (2012) IMA: an R package for high-throughput analysis of Illumina′s 450K Infinium methylation data. Bioinformatics 28: 729–730. 10.1093/bioinformatics/bts013 22253290 PMC3289916

[14] Hsieh CL (1994) Dependence of transcriptional repression on CpG methylation density. Mol Cell Biol 14: 5487–5494. 7518564 10.1128/mcb.14.8.5487-5494.1994 PMC359068

[15] Kirchner H, Osler ME, Krook A, Zierath JR (2013) Epigenetic flexibility in metabolic regulation: disease cause and prevention? Trends Cell Biol 23: 203–209. 10.1016/j.tcb.2012.11.008 23277089

[16] Baumer N, Tickenbrock L, Tschanter P, Lohmeyer L, Diederichs S, et al (2011) Inhibitor of cyclin-dependent kinase (CDK) interacting with cyclin A1 (INCA1) regulates proliferation and is repressed by oncogenic signaling. J Biol Chem 286: 28210–28222. 10.1074/jbc.M110.203471 21540187 PMC3151066

[17] Adams TD, Stroup AM, Gress RE, Adams KF, Calle EE, et al (2009) Cancer incidence and mortality after gastric bypass surgery. Obesity (Silver Spring) 17: 796–802. 10.1038/oby.2008.610 19148123 PMC2859193

[18] Arias AM, Bisschop PH, Ackermans MT, Nijpels G, Endert E, et al (2001) Aminophylline stimulates insulin secretion in patients with type 2 diabetes mellitus. Metabolism 50: 1030–1035. 10.1053/meta.2001.25800 11555834

[19] Rusing D, Muller CE, Verspohl EJ (2006) The impact of adenosine and A(2B) receptors on glucose homoeostasis. J Pharm Pharmacol 58: 1639–1645. 10.1211/jpp.58.12.0011 17331328

[20] Chapal J, Loubatieres-Mariani MM, Petit P, Roye M (1985) Evidence for an A2-subtype adenosine receptor on pancreatic glucagon secreting cells. Br J Pharmacol 86: 565–569. 10.1111/j.1476-5381.1985.tb08932.x 2998522 PMC1916740

[21] Barres R, Kirchner H, Rasmussen M, Yan J, Kantor FR, et al (2013) Weight loss after gastric bypass surgery in human obesity remodels promoter methylation. Cell Rep 3: 1020–1027. 10.1016/j.celrep.2013.03.018 23583180

