# Supplementary figures and images for: Roux-En Y Gastric Bypass Surgery Induces Genome-Wide Promoter-Specific Changes in DNA Methylation in Whole Blood of Obese Patients
Source: PLoS One. 2015 Feb 24;10(2):e0115186. doi: 10.1371/journal.pone.0115186 (PMC4340013; doi:10.1371/journal.pone.0115186)

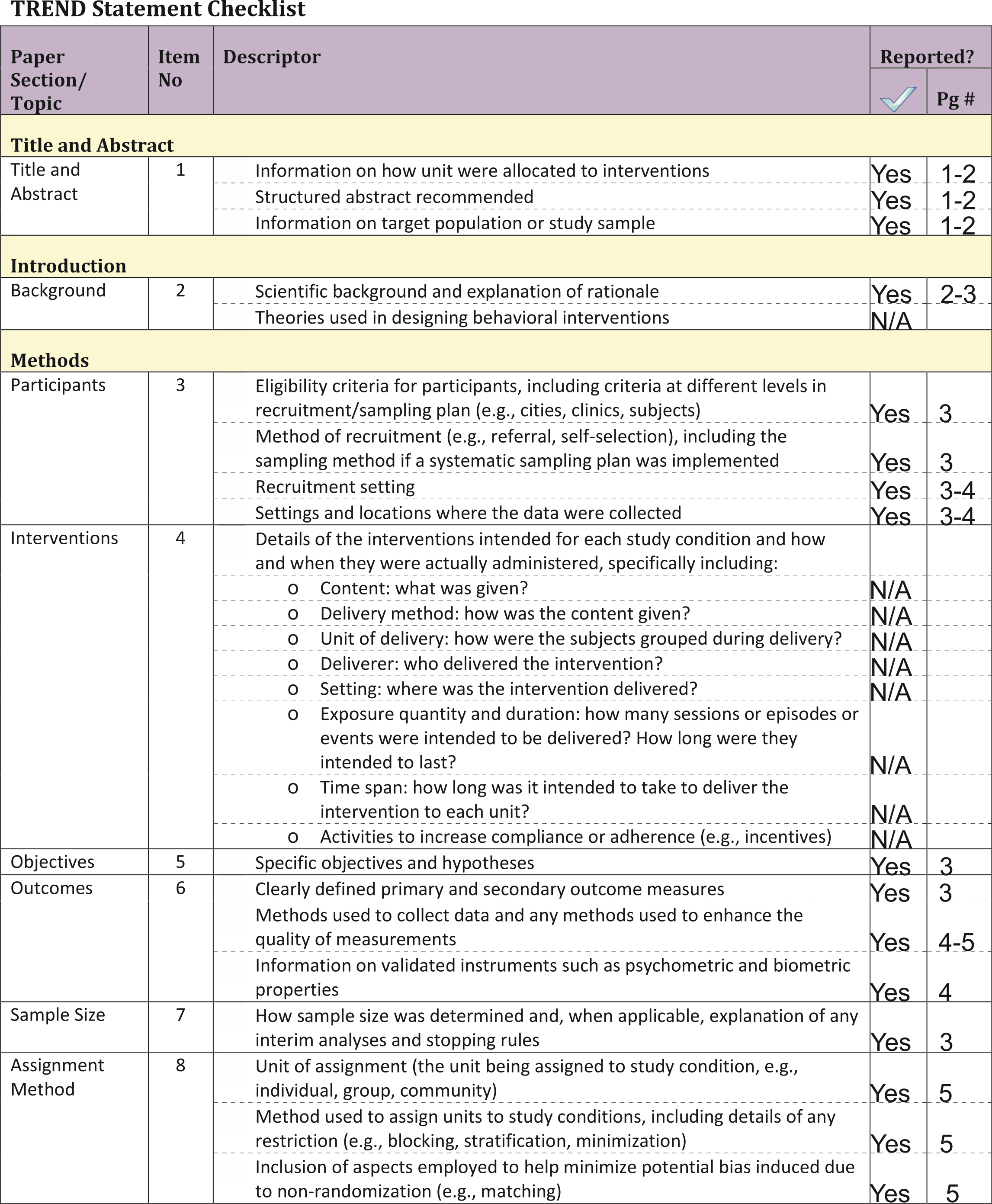

Supplement: S1 Trend Checklist — (TIFF) [file pone.0115186.s001.tiff]
